# The Expression of Stem Cell Marker LGR5 and Its Coexpression with Β-Catenin in Sporadic Colorectal Carcinoma and Adenoma: A Comparative Immunohistochemical Study

**DOI:** 10.3390/medicina59071233

**Published:** 2023-06-30

**Authors:** Eman Mohamed Ahmed, Abeer Said Farag, Mohammed S. Abdelwahed, Mehenaz Hanbazazh, Abdulhadi Samman, Diaa Ashmawy, Nageh Rady Abd-Elhameed, Mohamed Tharwat, Alyaa E. Othman, Taiseer Ahmed Shawky, Radwa Mohamed Attia, Adel Abdelwahid Ibrahim, Sherif Azzam, Mohammed E. A. Elhussiny, Mohamed Nasr, Suhaib Alsayed Naeem, Wagih M. Abd-Elhay, Ahmed Mohammad Ali Alfaifi, Abdulkarim Hasan

**Affiliations:** 1 Pathology Department, Faculty of Medicine for Girls, Al-Azhar University, Cairo 11884, Egypt; 2 Pathology Department, Faculty of Medicine, Al-Azhar University, Cairo 11884, Egypt; 3 Pathology Department, Faculty of Medicine, University of Jeddah, Jeddah 23218, Saudi Arabia; 4 Pathology Department, Faculty of Medicine, Al-Azhar University, Damietta 34517, Egypt; 5 Pathology Department, Faculty of Medicine, Al-Azhar University, Assiut 71524, Egypt; 6 Infectious Diseases Department, Faculty of Medicine, Suez Canal University, Ismailia 41552, Egypt; 7 Internal Medicine Department, Faculty of Medicine, Al-Azhar University, Cairo 11884, Egypt; 8 General Surgery Department, Faculty of Medicine for Girls, Al-Azhar University, Cairo 11884, Egypt; 9 Surgical Oncology Department, Faculty of Medicine, Al-Azhar University, Cairo 11884, Egypt; 10 Clinical Oncology Department, Faculty of Medicine, Al-Azhar University, Cairo 11884, Egypt; 11 Histology Department, Faculty of Medicine, Al-Azhar University, Cairo 11884, Egypt; 12 Laboratory Department, Aseer Central Hospital, Ministry of Health, Abha 62523, Saudi Arabia; 13 Prince Mishari bin Saud Hospital, Ministry of Health, Albahah 22888, Saudi Arabia

**Keywords:** colorectal cancer, cancer stem cell, LGR5, Β-catenin, immunohistochemistry

## Abstract

*Background*: LGR5 is one of the most important stem cell markers for colorectal cancer (CRC), as it potentiates Wnt/Β-catenin signaling. The well-characterized deregulation of Wnt/Β-catenin signaling that occurs during adenoma/carcinoma sequence in CRC renders LGR5 a hopeful therapeutic target. We assessed the immunohistochemical expression of LGR5 and Β-catenin in normal colonic and tumorous lesions with a clinicopathological correlation. *Methods*: Tissue blocks and clinical data of 50 selected cases were included: 8 from normal mucosa, 12 cases of adenoma, and 30 cases of CRC, where sections were cut and re-examined and the immunohistochemical technique was conducted using anti-LGR5 and anti-Β-catenin to measure the staining density. *Results*: There was no expression of LGR5 in normal mucosa compared to samples of adenoma and CRC samples. The association analysis showed that CRC specimens were more likely to have strong LGR5 and Β-catenin expressions than the other two groups (*p* = 0.048 and *p* < 0.001, respectively). Specimens with high-grade dysplastic adenoma were more likely to express moderate-to-strong expression of LGR5 and Β-catenin (*p* = 0.013 and *p* = 0.036, respectively). In contrast, there were no statistically significant associations between LGR5 and Β-catenin expression with grade and stage. *Conclusion*: These results suggest and support the possible role of LGR5 as a potential marker of cancer stem cells in sporadic colorectal carcinogenesis in addition to a prognostic value for LGR5 and Β-catenin in adenomatous lesions according to immunohistochemical expression density. A potential therapeutic role of LGR5 in CRC is suggested for future studies based on its role in pathogenesis.

## 1. Introduction

Colorectal cancer is the most prevalent malignancy of the gastrointestinal tract (GIT) and causes more than 610,000 deaths worldwide every year. It is the fourth most frequent cancer in Egypt, accounting for 4.35% of total malignancies and 15.87% of digestive system tumors. In the USA, CRC is the third most prevalent cancer in males after prostate and lung cancer and in females following breast and lung cancer [1,2,3,4].

The pathologist’s role in managing patients with CRC has expanded significantly from traditional histomorphologists to clinical consultants for oncologists, gastroenterologists, and colorectal surgeons with the rapid advancement of therapy in the era of personalized medicine [5]. 

Despite the continuous advances in the diagnosis of both primary and metastatic CRCs, the cure rates and the long-term survival in this common type of cancer are still limited [6]. Colon and rectal carcinomas usually start with abnormal noncancerous growth areas in the intestinal inner lining called colorectal polyps [7]. However, less than 10% of colorectal polyps have been seen transformed into invasive cancer [8]. This transition process occurs very slowly and gradually over 10 to 20 years or more with the increasing size of the raised polyp [9]. Later, the malignant cells may start to invade the lymphatic and muscular nodes before starting to spread to other organs such as liver, lungs, and bone [10]. The American Joint Committee of Cancer classified colon and rectal carcinomas into five stages; Stage 0 is characterized by abnormal colonic cells or polyps seen on the gut mucosa and is 100% curable with a surgical resection upon accurate and early detection. Resection of such polyps is also considered the treatment of choice for stages I–II and is usually associated with a 5-year survival rate in 36 to 74% of patients [6]. Unfortunately, in the advanced stages of CRC, the survival rate drops to 6% or less due to the high risk of tumor recurrence and distant metastasis to various organs [11]. 

Therefore, early and accurate diagnosis of CRC is crucial for preventing advanced stages and complications; however, this early diagnosis of CRC and colorectal polyps in symptomatic patients still remains a problem [12,13]. Moreover, the diagnosis of CRC is a complex process involving a typical sequence of events related to the initial consulting physician, the patient, and the healthcare system. Understanding this process, particularly the patient-related factors, including the genetic basis, is the first step in identifying the avoidable factors and reducing the serious effects of diagnostic delay on the tumor prognosis [13]. 

The carcinogenesis process of the CRC is a heterogeneous process of different sets of molecular changes influenced by various factors like age, gender, diet, smoking, alcohol intake, gastrointestinal microbiota, exposure to hazardous environmental agents, viral and bacterial infections, as well as the host immunity (host’s ability to respond to the recognized factors) [14,15]. In the last decade, some recent epidemiological studies have stated that obesity and lifestyle choices influence not only the risk of CRC development but also the morbidity and mortality which are associated with this cancer [16,17]. Immunosuppression is involved in the oncogenicity of CRC and also in the processes associated with tumor invasion and the metastasis of such carcinomas [18].

Genetic factors have been linked to the CRC carcinogenesis process, where the mutations of specific genes such as the oncogenes, tumor suppressor genes, and the genes involved in the deoxyribonucleic acid (DNA) repair process may lead to the initiation of colonic and rectal carcinomas [19,20]. 

The presence of chemotherapy-resistant cancer stem cells (CSCs) is thought to be one of the primary causes of tumor recurrence, which is a clinical nightmare and still a controversial process. These CSCs resist therapy abuse and re-establish the development of tumors subsequent to therapy action, so new and non-toxic cancer therapy that can lead to lasting clinical remissions is urgently needed [21]. 

Several signaling pathways, most notably the Wnt/β-catenin pathway, play an important role in maintaining the growth and functional integrity of CSC as well as in tumor initiation and growth. Therefore, a better understanding of the signaling mechanisms in CSC will aid in the development of new strategies for the treatment of such tumors [22]. The deregulation of the Wnt/β-catenin signaling pathway that is constitutively activated by genetic mutations into adenomatous polyposis coli (APC), or more rarely, β-catenin is an essential event to the early progression to CRC [23,24].

Most sporadic colorectal cancers are known to be initiated by activation mutations of the APC or β-catenin gene in the Wnt pathway, which results in β-catenin accumulation and constitutive transcriptional activation by the β-catenin/T-cell factor complex [25]. 

Leucine-rich, G-protein coupled receptor 5 (LGR5) is also a Wnt target gene, marking normal stem cells in various tissues, including the small and large intestines. During ordinary intestinal homeostasis, the expression of LGR5 is limited to the stem cell compartment located at the crypt base [26]. This expression of LGR5 is lost by the progeny of stem cells, as they migrate up through the amplifying zone of transit and undergo differentiation. Many studies have shown that CRC tissues maintain stem cell/progenitor hierarchies and LGR5 acts like CSC markers [27,28].

Research has further demonstrated that the LGR5 gene plays a role in the process of tumor progression, most likely due to this mutational activation of the Wnt/β-catenin pathway [29]. However, the expression of the immunohistochemical marker of LGR5 in sporadic CRCs and its clinical-pathological significance as well as its correlation with the expression of the β-catenin pathway in CRC are still not fully explored [30]. In this study, we aimed to evaluate the immunohistochemical expression of stem cell marker LGR5 and its co-expression with Β-catenin in sporadic colorectal carcinoma versus adenomas and to assess the correlation of their expression with the clinicopathological characters.

## 2. Materials and Methods

A cohort study was conducted on 50 selected cases of colorectal specimens (8 cases from normal mucosa taken in separate blocks from normal areas of colectomy specimens (group one), 12 cases of adenoma (group 2), and 30 cases of CRC (group 3)). The specimens were selected from the patients examined, diagnosed, and treated at Al-Azhar University Hospitals from the period between March 2019 and March 2020. Inclusion criteria include patients with sporadic CRC or colorectal adenoma during the study period with available demographic data, related clinical data, and anatomical pathology materials (tissue blocks and reports). Exclusion criteria include colitis-associated colorectal adenocarcinoma, cases with extensively necrotic tumors, and cases lacking agreement on the histologic diagnosis, grading, or scoring of the immunohistochemical markers.

### 2.1. Clinical (Internal Medicine) and Surgical Approach

The gastroenterologists from Al-Azhar University Hospitals clinically examined patients and proceeded with the colonoscopy procedure when indicated. Biopsies were taken from suspicious colorectal lesions for histopathological examination, and if polyps were found in the colon or rectum, a polypectomy was performed during the colonoscopy.

Surgical excision (radical surgery) with curative intent is considered the treatment of choice for the majority of CRCs. The basic surgical principles included removal of the major vascular pedicle feeding the tumor along with its related lymphatics, obtaining a tumor-free margin (at least a 5 cm margin of the adjacent normal bowel from both sides). Tumors in the transverse colon required transverse colectomy; no extended right colectomy was performed for the cecum, ascending colon, transverse colon, splenic flexure, or upper descending colon for the included cases. Descending and upper sigmoid colon lesions were treated with left hemicolectomy.

The clinical data of patients belonging to these samples were collected from the patients’ files and the clinicians in concern, and they included the age, gender, and tumor site. Specimens were sent for histopathology examination in formalin 10% fixative material.

### 2.2. Histopathology Examination

Multiple sections were cut; one was stained by hematoxylin and eosin for histopathological re-examination by the authors with a careful remote discussion using telepathology, while the other prepared sections were mounted on positive charged slides and immunostained with mouse monoclonal antibody against LGR5 and rabbit monoclonal antibody against Β-catenin for identification of stem cells. The histopathological examination was performed by at least three histopathologists separately, and only cases with agreement on the final diagnosis were included. Quality of the prepared sections and slides was assured before examination by the histologists, histotechnologists and/ot the histopathologist. 

### 2.3. Immunohistochemistry (IHC)

Immunohistochemical staining was carried out using Labelled Streptavidin-Biotin 2 System–Horseradish Peroxidase (LSAB2 System-HRP). The sections were deparaffinized in xylen and rehydrated in graded alcohol to distilled water. Sections were subjected to antigen retrieval by microwaving in 10 m of citrate buffer (sodium citrate, pH 6.0) for 30 min. Endogenous peroxidase activity was stopped (blocked) using 0.3% hydrogen peroxide in methanol for 15 min. At room temperature, the sections were incubated with anti-LGR5 (Santa Cruz Biotechnology Inc.; CA, USA) and anti-β-catenin (Transduction Laboratories, Lexington, KY, USA) at dilutions of 1:100 and 1:250, respectively, for 12 h. They were then stained by avidin–biotin and secondary antibodies according to the standard protocol and the manufacturer’s recommendations for each antibody. Sections were counterstained with hematoxylin stain, dehydrated, and mounted. We used a section of normal colonic epithelial cells as a positive control for LGR5 and normal oral epithelium for B catenin. The primary antibodies were replaced with PBS as a negative control for both markers.

### 2.4. Staining Evaluation

All antibody-stained sections were carefully examined and scored by three independent pathologists. LGR5-positive staining was indicated as a brown color in the cytoplasm with some membranous staining either in normal or tumor cells. Similarly, Β-catenin-positive staining was indicated as a brown coloration in the cell membrane in normal cells, and in the cytoplasm or in the nucleus of the tumor cells. For each immunostaining assay, the stain intensity was scaled as 0 (no stain), 1 (pale brown), 2 (brown), and 3 (dark brown). The quantity of immunoreactive cells was calculated and estimated as 0 (≤5% of total cells), 1 (6–25%), 2 (26–50%), 3 (51–75%), and 4 (>75%). Then, the raw data were converted to the IHS score by multiplying the intensity and the quantity scores for each antibody examined. An IHS of 9–12 was recorded as a strong immunoreactivity, 5–8 was considered moderate, 1–4 was considered weak, and 0 was scored as negative in agreement with previous studies [31].

### 2.5. Statistical Analysis

The collected data were computerized and statistically analyzed using SPSS program (version 23.0; IBM Corp, Armonk, NY, USA). Fisher’s Exact test and the Student’s *t* test were use to evaluate the association between histopathological features, grades, and stages (categorical and continuous, respectively) and the expression scores of IHC markers. *p* values of <0.05 and <0.01 indicate significant and highly significant results, respectively. 

## 3. Results

### 3.1. Histopathology and Patient Characteristics

Fifty archival paraffin blocks were used in the present study: 8 (16%) cases with a normal mucosa adjacent to the tumor, 12 (24%) cases of adenoma, and 30 (60%) cases of CRC. The clinical and histological criteria of the studied cases are summarized in Table 1. Histological types include tubular, villous, and tubulovillous adenomas, and the carcinoma cases include the conventional type and the mucinous adenocarcinoma. 

The adenocarcinomas were composed of anastomosed and ramified glandular structures with uneven lumens formed mainly of epithelial columnar cells with a cylindrical or pseudostratified aspect showing cytoplasm varied in abundancy within a homogeneous pattern and enlarged nuclei seen with hypochromic, vesicular, or one small nucleolus. The tumor glands presented empty lumens or lumens that were occupied by necrotic cells and cytoplasmic fragments.

Mucinous carcinomas are characterized by large collections of extra-cellular mucins within the extra-cellular environment, sometimes separated by tracts formed of fibroblasts pertaining to connective tissue cells and also inflammatory cells. The cellular components were greatly reduced when compared with the amount of mucin and formed of glands of irregular sizes, shapes, and dispositions. Some areas of the examined tumors appeared to be completely lacking glands and appeared to be formed of mucin lakes.

Tubular adenomas were given such a diagnosis if 75% of their tubular component consisted of at least round or oval glandular (tubular) profiles. For villous types, the percentage of villosity defines the diagnostic terminology as follows: 20–80% = tubulovillous adenoma; >80% = villous adenoma.

**Figure 1:**
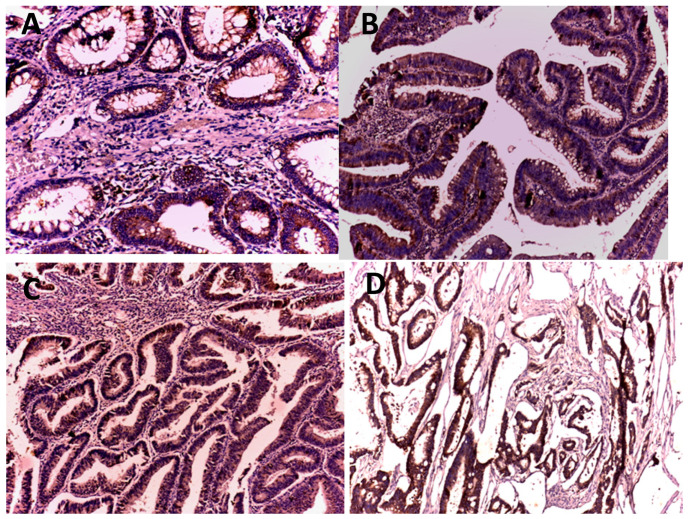
LGR5 Immunostaining: (**A**) tubular adenoma with weak membranous and focal cytoplasmic expression (×200); (**B**) villous adenoma with high-grade dysplasia showing strong cytoplasmic expression with membranous staining (×200); (**C**) grade I colorectal adenocarcinoma with weak cytoplasmic expression (×100); (**D**) mucinous adenocarcinoma showing strong cytoplasmic expression (×50).

The association analysis showed that CRC specimens were more likely to have strong LGR5 expression than the other two groups (normal and adenomatous groups) (*p* = 0.048). The majority of grade I cases showed moderate expression; however, the majority of grade II and III cases showed strong expression. Specimens of adenoma with high grades of dysplasia were more likely to express moderate-to-strong expression of LGR5. The expression was also increased with the stage of the tumor. On the contrary, no significant association between LGR5 expression and grade or stage of CRC was found (Table 2) (Figure 1).

### 3.2. Correlation between Β-Catenin Expression and the Clinico-Pathological Variables

**Figure 2:**
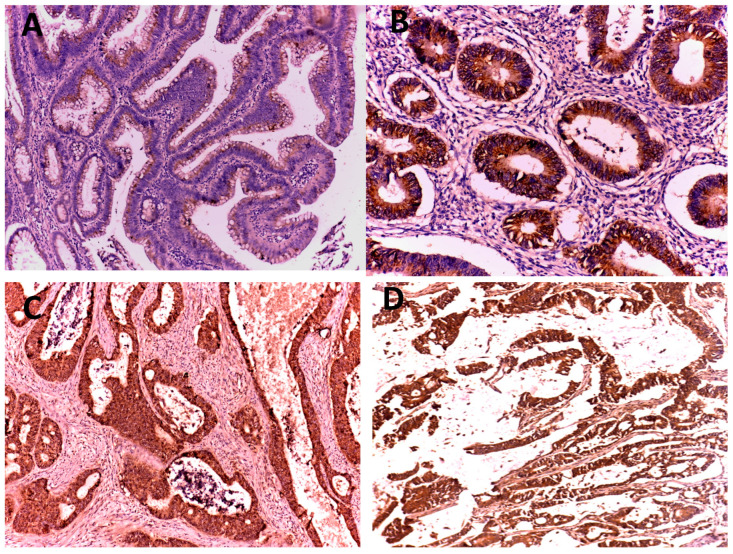
Β-catenin Immunostaining: (**A**) tubulovillous adenoma with low-grade dysplasia showing weak cytoplasmic expression with membranous staining (×200); (**B**) grade I colorectal adenocarcinoma with moderate cytoplasmic expression (×200); (**C**) grade II colorectal adenocarcinoma with strong cytoplasmic and nuclear expression (×100); (**D**) mucinous adenocarcinoma showing strong cytoplasmic and nuclear expression (×100).

Seven (87.50%) specimens of the normal mucosa adjacent to tumor had weak expression of Β-catenin. On the other hand, half of the adenoma specimens had a moderate expression of Β-catenin and half of the CRC specimens had a strong Β-catenin expression. The association analysis showed that CRC specimens were more likely to have strong Β-catenin expression than the other two groups (*p* < 0.001). On the contrary, there were no statistically significant associations between Β-catenin expression and the grade (*p* = 0.539) or stage of CRC (*p* = 0.309) (Table 3) (Figure 2). 

### 3.3. Correlation between Combined Expression and the Clinico-Pathological Variables

**Figure 3:**
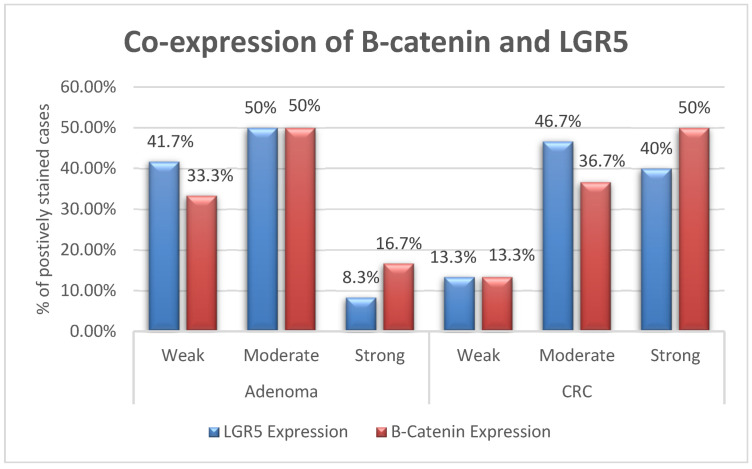
Comparison of LGR5 and Β-catenin expressions in the included specimens.

In the twelve cases of adenoma, the LGR5 and Β-catenin expressions were moderate in 6/12 (50%) of the cases. Strong expression was present in one case (8.3%) to LGR5 and two cases (16.7%) to Β-catenin, with a significant correlation between LGR5 and Β-catenin (*p* = 0.048). Similarly, 14 CRC cases (46.67%) show moderate expression for LGR5 and eleven cases (36.66) show moderate expression for Β-catenin. Strong expression was present in twelve cases (40%) for LGR5 and 15 cases (50%) for Β-catenin with significant correlation between LGR5 and Β-catenin (*p* = 0.038; Figure 3).

## 4. Discussion

The highest colonic cancer incidence rates are recorded in parts of Europe (e.g., in Slovenia, Hungary, Slovakia, Norway, and The Netherlands), Northern America, Australia/New Zealand, and Eastern Asia (Japan, the Republic of Korea and Singapore). Rates are elevated in Uruguay among genders, men and women, but Norway and Hungary rank first in males and females, respectively. Rectal cancer incidence rates reveal a similar regional distribution all over the world, although the highest rates are seen in Macedonia among females and in the Republic of Korea among males. In most geographic regions of Africa and in Southern Asia, rates of both colon and rectal cancer (colorectal cancer) tend to be low [2]. In Egypt, CRC is still a challenging diagnostic challenge but ranked the fourth most frequent cancer for 4.34% of total malignancies [2,32]. In 2020, more than 1.88 million new cases were diagnosed as CRC with around 900,000 deaths [33]. Individuals are routinely screened for CRC using either stool-based tests or endoscopic methods including flexible sigmoidoscopy and colonoscopy for early detection and to decrease the burden on individuals and communities [34,35], as almost all CRCs originate from adenomas or flat dysplasia then evolve into different morphologic gross patterns with invasion and expansion [35,36]. It is clear that the tumor prognosis of CRC is related to early diagnosis, for instance, the five-year survival following operation of CRC, diagnosed in the early stage is over 80% compared to 40% in the advanced stage [35]. Therefore, an early and deep understanding of the tumor pathogenesis is crucial for the improvement of colorectal cancer’s diagnostic markers especially for non-hereditary (sporadic) carcinomas. 

In colorectal cancers, CSC is suggested to be responsible for tumor initiation, growth, metastasis [37], tumor progression, recurrence [38], and resistance to chemotherapy [39]. Conventional cancer treatments that kill proliferating cells unsystematically are unsuccessful due to the survival of cancer stem cells. Therefore, therapies could be designed to target CSCs by inducing their differentiation or to eliminate inhibiting maintenance of stem-cell state [40].

In the present study, immunohistochemical analysis of stem cell marker LGR5 was carried in 12 cases of colorectal adenoma and 30 cases of colorectal carcinoma to determine its co-expression with Β-catenin as well as to assess the correlation of their expression with the clinicopathological characters.

Considering these findings related to LGR5, in normal cases LGR5 expression was negative in eight cases due to the paucity of immunopositive stained cells. 41.67% of adenoma cases showed weak expression and 50% showed moderate expression. Also, 46.67% of carcinoma cases showed moderate intensity, and 40% showed strong intensity compared with 8.33% of adenoma cases. The relation of LGR5 expression to the histopathological types of specimens was statistically significant (*p* = 0.048) because of more intense LGR5 immunoreactivity in CRC and adenoma than in normal mucosa. This suggests a possible role of LGR5 as a potential marker for cancer stem cells in colorectal carcinogenesis. These findings are in agreement with the results of Zeng et al. published in 2013 [41]. 

Regarding the grade of dysplasia in adenoma, we found that five cases of low-grade of dysplasia showed weak expression (83.33%), while in the high-grade dysplasia, five cases (83.3%) showed moderate expression. This difference is statistically significant (*p* = 0.013). This is similar to Baker et al. [42]. who mentioned that there was a generally higher level of LGR5 expression within regions of high-grade dysplastic lesions than the regions of low-grade dysplasia. This suggests an important role of LGR5 in the early molecular events in adenomatous lesions. This finding is in accordance with Dai et al. [43]. On the contrary, Takeda, et al. [25] found insignificant relationship between LGR5 expression and grade of dysplasia of adenoma.

Concerning the grade of CRC cases, there was no significant (*p* = 0.539) difference among the grades and intensity of LGR5 expression. However, the majority of grade I showed moderate expression and the majority of grade II and III showed strong expression. This finding is in accordance with Fan et al. [31] who suggests that LGR5 doesn’t have a role in the maintenance of the status of cell differentiation in carcinoma. On contrary, Gao et al. [44] found a significant relationship between LGR5 expression and tumor grade. 

No significant association between the stage of tumor and the LGR5 expression was detected in our study (*p* = 0.309). These results suggest that elevated immunohistochemical expression of LGR5 does not contribute to the progression of the tumor, and it could not be used as a potential unfavorable prognostic biomarker for CRC; however, the strength of expression was increased with the stage of tumor; 50% of stage II, 55.56% of stage III, and 60% of stage IV. Wu et al. [45] demonstrated that the high stage of CRC (stage IV) was associated with high level of LGR5 expression and high risk of poor prognosis.

Considering these findings related to Β-catenin, in our study Β-catenin expression was mainly confined within the membrane of normal colonic mucosa but progressively increased to include cell membrane, cytoplasm and/or the nucleus in adenoma and carcinoma. Beta catenin is a part of the WNT signaling pathway to be degraded by the degradation complex APC-Axin-GSK and it is a multifunctional protein complex, which is a component of cell-to-cell adhesion. In adenoma and carcinoma, beta-catenin degradations do not occur and cause increased accumulation of beta catenin in the cytoplasm. This means that membranous and cytoplasmic expression of Β-catenin increase with progression of carcinogenesis. The relation of Β-catenin expression to the histopathological types of specimens was statistically significant (*p* = 0.000). These findings are in agreement with the results of Fan et al. [31], Handjari et al. [46], and Wong et al. [47] who observed a significant increase in Β-catenin expression during the progression from normal epithelium to carcinoma.

As regards grade of dysplasia of adenoma, a significant relationship was found between Β-catenin expression and grade of dysplasia of adenoma (*p* = 0.036). This finding is in accordance with Dai et al. [43]. This suggests an important role of beta catenin in the early molecular events in adenomatous lesions. On the contrary, Silva et al. [48] found insignificant relationship between Β-catenin expression and the grade of dysplasia of adenoma. 

In our study, there was no significant association between the expression of Β-catenin and the grade of CRC (*p* = 0.539), this suggests that Β-catenin doesn’t have a role in maintenance of the status of cell differentiation in carcinoma. On the other hand, Kazem et al. [48] found a significant relationship between Β-catenin expression and tumor grade. 

According to stage, there was also no significant association between the expression of Β-catenin and stage (*p* = 0.309) suggesting that Β-catenin doesn’t have a role in the progression of the tumor. This is in contrast to Kazem et al. [49] and Fan et al. [31] who found that Β-catenin expression has a positive correlation with advanced tumor stage. 

In our study, a statistically significant relationship was found between LGR5 and Β-catenin expression (*p* = 0.038). This finding is in accordance with He et al. [30], Said et al. [50], Femia et al. [51] and Fan et al. [31]. On the contrary, Takeda et al. [25] found that LGR5 expression was not significantly associated with the expression of β-catenin. These results suggest potential involvement of LGR5 in colorectal carcinogenesis via WNT/Β-catenin pathway. 

Due to its key role in tumorigenesis, LGR5 expression has shown a significant correlation with the survival of patients according to many previously published studies which explored unfavorable prognostic results in CRC [45,52,53,54]. However, contrary results have been suggested by certain reports for the correlation between LGR5 staining scores and the prognosis of CRC [5], for example, a genetic study published in 2012 by Ziskin et al. [55] and an immunohistochemical study published in 2019 by Shekarriz et al. [56] reported that LGR5 expression was not seen significantly associated with the aggressiveness of colorectal tumors. The limitations of our study include lack of correlation with other assays for LGR5 and Β-catenin assessment in addition to the number of the studied cases which is recommended to be increased in the future studies focusing on LGR5 expression in normal intestinal mucosa cases and also studying the clinical differences such as therapy response and survival.

## 5. Conclusions

Our study suggests a possible role for LGR5 as a potential marker of cancer stem cells in colorectal carcinogenesis in sporadic CRC and in the early molecular events in adenomatous lesions but does not have a role in the maintenance of the status of cell differentiation or in the progression of the CRC, however, expression density was significantly associated with the adenomatous grade and a significant difference was shown between adenoma and carcinoma. β-catenin expression also positively correlates with LGR5 over-expression, suggesting the potential involvement of combining LGR5 and β-catenin immunohistochemical markers and degree of expression in the colorectal diagnostic panel for adenoma and carcinoma.

## Figures and Tables

**Table 1:** Clinico-histological characteristics of the enrolled patients.

		Count	N (%)
Age	Mean ± SD	49.64 ± 7.96	
Sex	Male	30	60%
	Female	20	40%
Tumor histology	Normal	8	16%
	Adenoma	12	24%
	CRC	30	60%
Histopathological types of adenoma	Tubular	2	16.67%
	Tubulovillous	6	50%
	Villous	4	33.33%
Histopathological types of CRC	Conventional type adenocarcinoma	27	90%
	Mucinous adenocarcinoma	3	10.00%
Stage of CRC	Stage I	8	26.7%
	Stage II	8	26.7%
	Stage III	9	30%
	Stage IV	5	16.7%
Grade of CRC	Grade I	15	50%
	Grade II	10	33.3%
	Grade III	5	16.7%

**Table 2:** Correlation between LGR5 expression and the histopathological variables.

Colorectal Studied Cases		No.	%	Weak		Moderate		Strong		*p*-Value
				No.	%	No.	%	No.	%	
Histopathological types	Normal	8	16%	0	0.0%	0	0.0%	0	0.0%	0.048
	Adenoma	12	24%	5	41.67%	6	50.00%	1	8.33%	
	Carcinoma	30	60.00%	4	13.33%	14	46.67%	12	40.00%	
Grade of Dysplasia In Cases of Adenoma	High dysplasia	6	50%	0	0.00%	5	83.33%	1	16.67%	0.013
	Low dysplasia	6	50%	5	83.33%	1	16.67%	0	0.00%	
Grade of CRC	Grade I	15	50%	3	20.00%	8	53.33%	4	26.67%	0.566
	Grade II	10	33.33%	1	10.00%	4	40.00%	5	50.00%	
	Grade III	5	16.7%	0	0.00%	2	40.00%	3	60.00%	
Stage of CRC	Stage I	8	26.7%	3	37.50%	5	62.50%	0	0.00%	0.107
	Stage II	8	26.7%	1	12.50%	3	37.50%	4	50.00%	
	Stage III	9	30%	0	0.00%	4	44.44%	5	55.56%	
	Stage IV	5	16.7%	0	0.00%	2	40.00%	3	60.00%	

**Table 3:** Correlation between Β-catenin expression and the histopathological variables.

Colorectal Studied Cases		NO.	%	Weak		Moderate		Strong		*p*-Value
				No.	%	No.	%	No.	%	
Histopathological type	Normal	8	6%	7	87.50%	1	12.50%	0	0.00%	0.000
	Adenoma	12	4%	4	33.33%	6	50.00%	2	16.67%	
	Carcinoma	30	0%	4	13.33%	11	36.67%	15	50.00%	
Grade of dysplasia in adenoma	High	6	0%	0	0.00%	4	66.67%	2	33.33%	0.036
	Low	6	50%	4	66.67%	2	33.33%	0	0.00%	
Grade of colorectal adenocarcinoma	Grade I	15	0%	2	13.33%	7	46.67%	6	40.00%	0.539
	Grade II	10	33.3%	2	20.00%	3	30.00%	5	50.00%	
	Grade III	5	16.7%	0	0.00%	1	20.00%	4	80.00%	
Stage of colorectal adenocarcinoma	Stage I	8	26.7%	2	25.00%	5	62.50%	1	12.50%	0.309
	Stage II	8	26.7%	1	12.50%	2	25.00%	5	62.50%	
	Stage III	9	30%	1	11.11%	3	33.33%	5	55.56%	
	Stage IV	5	16.7%	0	0.00%	1	20.00%	4	80.00%	

## Data Availability

Data are available upon request to the corresponding author.

## References

[1] Thanikachalam K., Khan G. (2019). Colorectal Cancer and Nutrition. Nutrients.

[2] Bray F., Ferlay J., Soerjomataram I., Siegel R.L., Torre L.A., Jemal A. (2018). Global cancer statistics 2018: GLOBOCAN estimates of incidence and mortality worldwide for 36 cancers in 185 countries. CA Cancer J. Clin..

[3] Alteri R., Brooks D., Cokkinide V. (2013). Colorectal Cancer Facts & Figures.

[4] Mokhtar N., Adel I., Goda I. (2007). Cancer Pathology Registry 2003–2004 and Time Trend Analysis.

[5] Fleming M., Ravula S., Tatishchev S.F., Wang H.L. (2012). Colorectal carcinoma: Pathologic aspects. J. Gastrointest. Oncol..

[6] Haider M., Zaki K.Z., El Hamshary M.R., Hussain Z., Orive G., Ibrahim H.O. (2022). Polymeric nanocarriers: A promising tool for early diagnosis and efficient treatment of colorectal cancer. J. Adv. Res..

[7] Siegel R.L., Miller K.D., Jemal A. (2018). Cancer statistics, 2018. CA A Cancer J. Clin..

[8] Risio M. (2010). Reprint of: The natural history of adenomas. Best Pract. Res. Clin. Gastroenterol..

[9] Pickhardt P.J., Kim D.H., Pooler B.D., Hinshaw J.L., Barlow D., Jensen D., Reichelderfer M., Cash B.D. (2013). Assessment of volumetric growth rates of small colorectal polyps with CT colonography: A longitudinal study of natural history. Lancet Oncol..

[10] You X., Kang Y., Hollett G., Chen X., Zhao W., Gu Z., Wu J. (2016). Polymeric nanoparticles for colon cancer therapy: Overview and perspectives. J. Mater. Chem. B..

[11] Cisterna B.A., Kamaly N., Choi W.I., Tavakkoli A., Farokhzad O.C., Vilos C. (2016). Targeted nanoparticles for colorectal cancer. Nanomedicine.

[12] Vega P., Valentín F., Cubiella J. (2015). Colorectal cancer diagnosis: Pitfalls and opportunities. World J. Gastrointest. Oncol..

[13] Langenbach M.R., Schmidt J., Neumann J., Zirngibl H. (2003). Delay in treatment of colorectal cancer: Multifactorial problem. World J. Surg..

[14] O’Keefe S.J.D. (2016). Diet, microorganisms and their metabolites, and colon cancer. Nat. Rev. Gastroenterol. Hepatol..

[15] Ilie D.S., Mitroi G., Păun I., Ţenea-Cojan T.Ş., Neamţu C., Totolici B.D., Sapalidis K., Mogoantă S.Ş., Murea A. (2021). Pathological and immunohistochemical study of colon cancer. Evaluation of markers for colon cancer stem cells. Rom. J. Morphol. Embryol..

[16] Park J., Morley T.S., Kim M., Clegg D.J., Scherer P.E. (2014). Obesity and cancer—Mechanisms underlying tumour progression and recurrence. Nat. Rev. Endocrinol..

[17] Schwartz B., Yehuda-Shnaidman E. (2014). Putative role of adipose tissue in growth and metabolism of colon cancer cells. Front. Oncol..

[18] Grady W.M., Carethers J.M. (2008). Genomic and epigenetic instability in colorectal cancer pathogenesis. Gastroenterology.

[19] Mármol I., Sánchez-de-Diego C., Pradilla Dieste A., Cerrada E., Rodriguez Yoldi M.J. (2017). Colorectal carcinoma: A general overview and future perspectives in colorectal cancer. Int. J. Mol. Sci..

[20] Schwitalla S., Fingerle A.A., Cammareri P., Nebelsiek T., Göktuna S.I., Ziegler P.K., Canli O., Heijmans J., Huels D.J., Moreaux G. (2013). Intestinal tumorigenesis initiated by dedifferentiation and acquisition of stem-cell-like properties. Cell.

[21] Phi L.T.H., Sari I.N., Yang Y.-G., Lee S.-H., Jun N., Kim K.S., Lee Y.K., Kwon H.Y. (2018). Cancer Stem Cells (CSCs) in Drug Resistance and their Therapeutic Implications in Cancer Treatment. Stem Cells Int..

[22] Roy S., Majumdar A.P. (2012). Signaling in colon cancer stem cells. J. Mol. Signal..

[23] Zhan T., Rindtorff N., Boutros M. (2017). Wnt signaling in cancer. Oncogene.

[24] White B.D., Chien A.J., Dawson D.W. (2012). Dysregulation of Wnt/β-Catenin Signaling in Gastrointestinal Cancers. Gastroenterology.

[25] Takeda K., Kinoshita I., Shimizu Y., Matsuno Y., Shichinohe T., Dosaka-Akita H. (2011). Expression of LGR5, an intestinal stem cell marker, during each stage of colorectal tumorigenesis. Anticancer. Res..

[26] Barker N., Tan S., Clevers H. (2013). Lgr proteins in epithelial stem cell biology. Development.

[27] Merlos-Suárez A., Barriga F.M., Jung P., Iglesias M., Céspedes M.V., Rossell D., Sevillano M., Hernando-Momblona X., da Silva-Diz V., Muñoz P. (2011). The Intestinal Stem Cell Signature Identifies Colorectal Cancer Stem Cells and Predicts Disease Relapse. Cell Stem Cell.

[28] Barker N., Ridgway R.A., van Es J.H., van de Wetering M., Begthel H., van den Born M., Danenberg E., Clarke A.R., Sansom O.J., Clevers H. (2009). Crypt stem cells as the cells-of-origin of intestinal cancer. Nature.

[29] Barker N., van Es J.H., Kuipers J., Kujala P., van den Born M., Cozijnsen M., Haegebarth A., Korving J., Begthel H., Peters P.J. (2007). Identification of stem cells in small intestine and colon by marker gene Lgr5. Nature.

[30] He S., Zhou H., Zhu X., Hu S., Fei M., Wan D., Gu W., Yang X., Shi D., Zhou J. (2014). Expression of Lgr5, a marker of intestinal stem cells, in colorectal cancer and its clinicopathological significance. Biomed. Pharmacother..

[31] Fan X.-S., Wu H.-Y., Yu H.-P., Zhou Q., Zhang Y.-F., Huang Q. (2010). Expression of Lgr5 in human colorectal carcinogenesis and its potential correlation with β-catenin. Int. J. Color. Dis..

[32] Ghoniem I., Elghamry F.G., Abobakr A.M. (2022). Diagnostic value of plasma M2-pyruvate kinase in Egyptian patients with colorectal cancer. Al-Azhar Int. Med. J..

[33] Xi Y., Xu P. (2021). Global colorectal cancer burden in 2020 and projections to 2040. Transl. Oncol..

[34] Kopel J., Ristic B., Brower G.L., Goyal H. (2022). Global Impact of COVID-19 on Colorectal Cancer Screening: Current Insights and Future Directions. Medicina.

[35] Zhang Y.L., Zhang Z.S., Wu B.P., Zhou D.Y. (2002). Early diagnosis for colorectal cancer in China. World J. Gastroenterol..

[36] Dawood G. (2022). Gastrointestinal System. Color Atlas of Human Gross Pathology.

[37] Kemper K., Prasetyanti P.R., De Lau W., Rodermond H., Clevers H., Medema J.P. (2012). Monoclonal antibodies against Lgr5 identify human colorectal cancer stem cells. Stem Cell.

[38] Chen X., Wei B., Han X., Zheng Z., Huang J., Liu J., Huang Y., Wei H. (2014). LGR5 is required for the maintenance of spheroid-derived colon cancer stem cells. Int. J. Mol. Med..

[39] Espersen M.L.M., Olsen J., Linnemann D., Høgdall E., Troelsen J.T. (2015). Clinical Implications of Intestinal Stem Cell Markers in Colorectal Cancer. Clin. Color. Cancer.

[40] AL-Hajj M. (2007). Cancer stem cells and oncology therapeutics. Curr. Opin. Oncol..

[41] Wu J., Xie N., Xie K., Zeng J., Cheng L., Lei Y., Liu Y., Song L., Dong D., Chen Y. (2013). GPR48, a poor prognostic factor, promotes tumor metastasis and activates β-catenin/TCF signaling in colorectal cancer. Carcinogenesis.

[42] Baker A.-M., Graham T.A., Elia G., Wright N.A., Rodriguez-Justo M. (2015). Characterization of LGR5 stem cells in colorectal adenomas and carcinomas. Sci. Rep..

[43] Dai X., Wang L., Zhang L., Han Y., Yang G., Li L. (2012). The expression and mutation of β-catenin in colorectal traditional serrated adenomas. Indian J. Pathol. Microbiol..

[44] Gao F.-J., Chen J.-Y., Wu H.-Y., Shi J., Chen M., Fan X.-S., Huang Q. (2014). Lgr5 over-expression is positively related to the tumor progression and HER2 expression in stage pTNM IV colorectal cancer. Int. J. Clin. Exp. Pathol..

[45] Wu S.X., Xi Q.H., Chen L. (2012). LGR5 is a potential marker of colorectal carcinoma stem cells that correlates with patient survival. World J. Surg. Oncol..

[46] Handjari R.D., Abineno P., Siregar B. (2011). Subcellular localization of Β-catenin in colorectal non neoplastic and neoplastic lesions. Makara Kesehatan.

[47] Wong S.C.C., Lo E.S.F., Lee K.C., Chan J.K., Hsiao W.W. (2004). Prognostic and Diagnostic Significance of β-Catenin Nuclear Immunostaining in Colorectal Cancer. Clin. Cancer Res..

[48] Silva S.R.M., Matos D., Waitzberg A.F., Artigiani R., Saad S.S. (2011). Study of APC and β-catenin protein expression in polyps and colorectal adenocarcinoma. Appl. Cancer Res..

[49] Kazem A., El Sayed K., El Kerm Y. (2014). Prognostic significance of COX-2 and β-catenin in colorectal carcinoma. Alexandria.

[50] Said N., Mangoud A., Mostafa S., Yehia M., El-Aziz A. (2017). EXPRESSION OF LGR5 AND BETA-CATENIN IN BENIGN AND MALIGNANT COLORECTAL LESIONS IN ZAGAZIG UNIVERSITY HOSPITAL. Zagazig Univ. Med. J..

[51] Femia A.P., Dolara P., Salvadori M., Caderni G. (2013). Expression of LGR-5, MSI-1 and DCAMKL-1, putative stem cell markers, in the early phases of 1, 2-dimethylhydrazine-induced rat colon carcinogenesis: Correlation with nuclear β-catenin. BMC Cancer.

[52] Liu Z., Dai W., Jiang L., Cheng Y. (2014). Over-expression of LGR5 correlates with poor survival of colon cancer in mice as well as in patients. Neoplasma.

[53] Morsy H., Gaballah A., Samir M., Nakundi V., Shamseya M., Mahrous H., Ghazal A., Hashish M., Arafat W. (2021). LGR5, HES1 and ATOH1 in Young Rectal Cancer Patients in Egyptian. Asian Pac. J. Cancer Prev..

[54] Chen Q., Zhang X., Li W.M., Ji Y.Q., Cao H.Z., Zheng P. (2014). Prognostic value of LGR5 in colorectal cancer: A meta-analysis. PLoS ONE.

[55] Ziskin J.L., Dunlap D., Yaylaoglu M., Fodor I.K., Forrest W.F., Patel R., Ge N., Hutchins G.G., Pine J.K., Quirke P. (2013). In situ validation of an intestinal stem cell signature in colorectal cancer. Gut.

[56] Shekarriz R., Montazer F., Alizadeh-Navaei R. (2019). Overexpression of cancer stem cell marker Lgr5 in colorectal cancer patients and association with clinicopathological findings. Casp. J. Intern. Med..

